# Surgery

**Published:** 1915-12

**Authors:** A. Rendle Short


### Progress of the Medical Sciences.

### SURGERY.

**Sepsis and Antisepsis in Wounds.**—As was only to be expected, one object of medical research during the war has been to ascertain the nature of infection that may follow a gunshot injury, and the best means of prevention and treatment. Such studies will have a permanent value in civil surgery also, because the essential features of gunshot wounds, namely a long narrow track infected by earth or fragments of clothing, and containing lodged foreign bodies or pieces of splintered bone, may be reproduced by the injuries of peace as well as of war.

*Nature of the Infection.*—Fleming[1] examined bacteriologically a large number of wounds at a base hospital at

[1] *Lancet*, 1915, ii. 638.

Boulogne. He found that the micro-organisms present were by no means the same as those found in wounds caused by, shall we say, industrial accidents in England, which are not infected either by earth or clothing.

Early, one to seven days, Fleming found *Bacillus aerogenes capsulatus*, 90 per cent.; *streptococci*, 90 per cent.; *staphylococci*, 30 per cent.; *bacillus tetani*, 15 per cent.; coliform bacteria, B. *x* and *y*, and other indeterminate organisms, occasionally. Later the bacilli die out, and the wounds contain principally streptococci.

All these bacilli are probably of fæcal origin, either human or animal. According to Sir Almroth Wright, the streptococcus is also fæcal, the *enterococcus* of French bacteriologists. Needless to say, it does not follow that a patient with *B. aerogenes capsulatus* in the wound necessarily develops gas gangrene; a main determining factor in bringing about that complication appears to be lack of drainage. Nor does infection with *B. tetani* necessarily produce lock-jaw. Up to December last about 1 in 200 of the wounds led to tetanus, but now that antitoxin is so frequently given as a prophylactic the disease has become quite uncommon. Last February the proportion developing gas gangrene was put at 2 to 3 per cent. (Sir W. Watson Cheyne).

*Antiseptic Treatment of Wounds.*—A number of interesting researches have been published as to the relative value of various antiseptic and other applications.

Delorme,[1] speaking for the French school of military surgery, advocates as a first application, immediately after infliction of the wound, either a single brush of tincture of iodine, or a dry sterile gauze dressing, not to be handled directly by the wounded man or attendant. Afterwards hydrogen peroxide is to be the main stand-by in the treatment of all infected wounds.

Sir Watson Cheyne,[2] with Fleet-Surgeon Bassett Smith and Mr. Edmunds, acting as a Naval Committee, have studied the penetrative power of various antiseptics through gelatinous media, such as agar and blood-clot. The method was to put an antiseptic paste at the bottom of a Petri dish, pour in agar a quarter inch thick, sow it with staphylococci or various sporing organisms, incubate, and observe whether the antiseptic under the agar sufficed to check growth on the surface. They found that iodine was quite ineffective; the best penetrating antiseptics were carbolic acid and the cresols (liq. cresol saponatus, lysol, etc.). They therefore advise a paste of 20 per cent. carbolic acid in lanoline (six parts) and white wax (one part), to be applied to the wound as soon as possible after infliction.

[1] Delorme, *War Surgery*. [2] *Lancet*, 1915, i. 419.

The paste is, of course, more lasting than a lotion, and it is not irritating. A few cases are recorded in which it seemed to do well.

In a later communication they report that a mixture of salicylic and boric acids injected into guinea-pigs along with a highly infected earth containing bacilli of tetanus and gas gangrene quite prevented those diseases.

Sir Watson Cheyne reminds us that the introduction by Lister of crude phenol, as an application for dirty wounds, went far towards abolishing tetanus, which had previously been common in his wards.

Mr. Bond [1] has dealt experimentally with the objection that strong antiseptics delay healing. He has systematically painted wounds in the course of operation for hernia, varicose veins, etc., with pure carbolic, tincture of iodine, 1 in 20 carbolic, or 1 in 1,000 mercuric perchloride, and found that there was no difference in the appearance and rate of healing as compared with a wound in which no antiseptics were used. One may comment, however, that these wounds are much smaller than many of those inflicted in war.

Mr. K. Taylor [2] publishes some experiments with quinine in the prevention of gas gangrene in guinea-pigs. It is far more powerful against *B. aerogenes capsulatus* than carbolic acid *in vitro*, and whereas apart from its use all the animals inoculated died of gas gangrene, with it 59 per cent. recovered. He suggests a 1 per cent. solution, which is non-toxic.

What promises to be a very valuable new antiseptic has been described almost simultaneously by several workers; in England by Professor Lorrain Smith [3] and coadjutors. This is hypochlorous acid, obtained by mixing boric acid and bleaching powder. It may be used in powder form, called "eupad," or as a solution (25 grm. to the litre), called "eusol." It must be used fresh. Its great virtues are that it is cheap, non-toxic, not usually irritating, and far more effectual than 1 in 20 carbolic; it rapidly kills spores of tetanus and other organisms. I have found eusol very valuable in a few cases of recent dirty wounds. Surgeon H. E. R. Edwards [4] reports very favourably on its use in the Navy on H.M.S. *Lion*.

Sir Almroth Wright [5] teaches that antiseptics are of little or no value in the treatment of infected wounds. They do not penetrate into pus, clot or living tissue to any useful extent, and only kill the germs in the free discharge which is going down the drain anyhow. Possibly an exception must be made in favour

[1] *Brit. M. J.*, 1915, i. 405. [2] *Lancet*, 1915, ii. 538.
[3] *Brit. M. J.*, 1915, July 24th.
[4] *Journ. Royal Naval Med. Service*, 1915, October.
[5] *Brit. M. J.*, 1915, ii. 629, 670, 717.

of such drugs as salvarsan and optoquin, which do not coagulate albumin, and have a special chemical affinity for particular organisms. He advises, (1) in the first-aid station, that bleeding should be stopped, splints applied, the wound cleaned, and a vaccine given (streptococci, staphylococci, B. aerogenes capsulatus). (2) In the field ambulance, extract foreign bodies, and give anti-tetanic serum. Secure efficient drainage, and apply hypertonic saline solution (5 per cent., with 0.5 per cent. of sodium citrate) by wicks of gauze carried down into the wound, and covered with pads soaked in the same, with a tabloid or two of salt in the dressing. Protect the skin with vaseline. (3) At the base hospital, irrigate with hypertonic saline till the wound is healthy, then use normal saline instead. If cellulitis or gas gangrene develop, or pocketing of pus occurs, make incisions, and apply hypertonic saline. If a joint is wounded irrigate with normal saline from the first.

The function of the hypertonic saline is to draw out lymph through the infected area, and so to bring its bactericidal properties into play; the citrate is to stop clotting. The strong saline, however, kills the leucocytes, therefore when the position is won, but not consolidated, we change over to normal saline to bring up the white blood corpuscles into the scene of action.

A few words may be added by way of summary. Many small wounds will heal by first intention under any sensible line of treatment. The main value of antiseptics is within the first forty-eight hours; after that it is doubtful if they do much good. Whatever method of treatment is adopted, removal of dirt or clothing, and the provision of free drainage are supremely important. Of antiseptics in the early stage (under forty-eight hours), eusol, the salicyl-boric mixture or quinine are probably best, because these kill spores as well as pyogenic cocci. Later, the competition lies between hypertonic saline and hydrogen peroxide. If gas gangrene threatens, injection of oxygen into the tissues around the wound is valuable.

A. Rendle Short.

